# Polymeric nanoparticle-encapsulated curcumin ("nanocurcumin"): a novel strategy for human cancer therapy

**DOI:** 10.1186/1477-3155-5-3

**Published:** 2007-04-17

**Authors:** Savita Bisht, Georg Feldmann, Sheetal Soni, Rajani Ravi, Collins Karikar, Amarnath Maitra, Anirban Maitra

**Affiliations:** 1 The Sol Goldman Pancreatic Cancer Research Center, Department of Pathology, Johns Hopkins University School of Medicine, Baltimore, Maryland, USA; 2 Department of Oncology, Johns Hopkins University School of Medicine, Baltimore, Maryland, USA; 3 Department of Chemistry, University of Delhi, Delhi, India

## Abstract

**Background:**

Curcumin, a yellow polyphenol extracted from the rhizome of turmeric (*Curcuma longa*), has potent anti-cancer properties as demonstrated in a plethora of human cancer cell line and animal carcinogenesis models. Nevertheless, widespread clinical application of this relatively efficacious agent in cancer and other diseases has been limited due to poor aqueous solubility, and consequently, minimal systemic bioavailability. Nanoparticle-based drug delivery approaches have the potential for rendering hydrophobic agents like curcumin dispersible in aqueous media, thus circumventing the pitfalls of poor solubility.

**Results:**

We have synthesized polymeric nanoparticle encapsulated formulation of curcumin – nanocurcumin – utilizing the micellar aggregates of cross-linked and random copolymers of N-isopropylacrylamide (NIPAAM), with N-vinyl-2-pyrrolidone (VP) and poly(ethyleneglycol)monoacrylate (PEG-A). Physico-chemical characterization of the polymeric nanoparticles by dynamic laser light scattering and transmission electron microscopy confirms a narrow size distribution in the 50 nm range. Nanocurcumin, unlike free curcumin, is readily dispersed in aqueous media. Nanocurcumin demonstrates comparable *in vitro *therapeutic efficacy to free curcumin against a panel of human pancreatic cancer cell lines, as assessed by cell viability and clonogenicity assays in soft agar. Further, nanocurcumin's mechanisms of action on pancreatic cancer cells mirror that of free curcumin, including induction of cellular apoptosis, blockade of nuclear factor kappa B (NFκB) activation, and downregulation of steady state levels of multiple pro-inflammatory cytokines (IL-6, IL-8, and TNFα).

**Conclusion:**

Nanocurcumin provides an opportunity to expand the clinical repertoire of this efficacious agent by enabling ready aqueous dispersion. Future studies utilizing nanocurcumin are warranted in pre-clinical *in vivo *models of cancer and other diseases that might benefit from the effects of curcumin.

## Background

Curcumin or diferuloylmethane is a yellow polyphenol extracted from the rhizome of turmeric (*Curcuma longa*), a plant grown in tropical Southeast Asia [1]. For centuries, turmeric has been used as a spice and coloring agent in Indian food, as well as a therapeutic agent in traditional Indian medicine. Enthusiasm for curcumin as an anti-cancer agent evolved based on the wealth of epidemiological evidence suggesting a correlation between dietary turmeric and low incidence of gastrointestinal mucosal cancers [2,3]. A plethora of experimental data has unequivocally established that free curcumin induces cell cycle arrest and/or apoptosis in human cancer cell lines derived from a variety of solid tumors including colorectal, lung, breast, pancreatic and prostate carcinoma, amongst others [4-12]. In addition to a potential application in cancer therapy, studies in numerous experimental (chemical) carcinogenesis models [13-17], and more recently in a clinical trial performed in patients with familial adenomatous polyposis [18], have confirmed that curcumin can also ameliorate the progression to cancer in a variety of organ sites, reiterating this agent's potential as a tool for chemoprevention.

Despite the considerable promise that curcumin is an efficacious and safe compound for cancer therapy and chemoprevention, it has by no means been embraced by the cancer community as a "panacea for all ills". The single most important reason for this reticence has been the reduced bioavailability of orally administered curcumin, such that therapeutic effects are essentially limited to the tubular lower GI tract (i.e., colorectum) [19,20]. For example, in a Phase I clinical trial, patients with hepatic colorectal cancer metastases were administered 3600 mg of oral curcumin daily, and levels of curcumin and its metabolites measured by HPLC in portal and peripheral blood [21]. Curcumin was poorly available following oral administration, with low nanomolar levels of the parent compound and its glucuronide and sulphate conjugates found in the peripheral or portal circulation. In another Phase I study, patients were required to partake 8000 mg of free curcumin orally per day, in order to achieve detectable systemic levels; beyond 8 grams, the bulky volume of the drug was unacceptable to patients [22]. A third human Phase I trial involving curcumin dose escalation found no trace of this compound at doses of 500–8,000 mg/day, and only trace amounts in a minority of patients at 10–12 grams of curcumin intake per day [23]. The development of a delivery system that can enable parenteral administration of curcumin in an aqueous phase medium will significantly harness the potential of this promising anti-cancer agent in the clinical arena.

We report the synthesis, physico-chemical characterization, and cancer-related application of a nanoparticle-encapsulated formulation of curcumin, "nanocurcumin". Cross-linked polymeric nanoparticles with a hydrophobic core and a hydrophilic shell were used for encapsulation of curcumin, generating drug-encapsulated nanoparticles consistently in size less than 100 nm.

## Results and discussion

### Synthesis and detailed physico-chemical characterization of NIPAAM/VP/PEG-A copolymeric nanoparticles (FT-IR, ^1^H-NMR, DLS, TEM and Release Kinetics)

**Figure 1:**
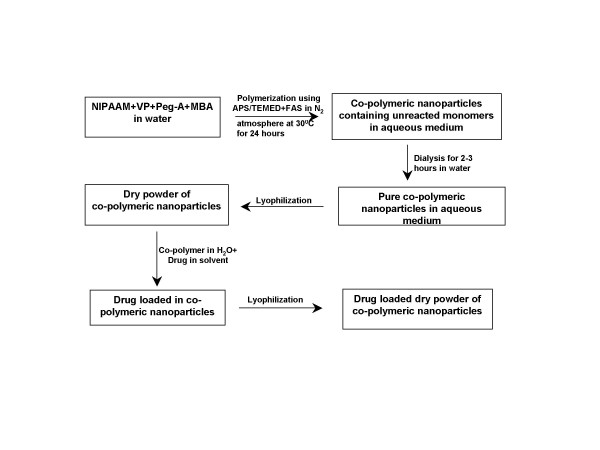
**Synthesis strategy for NIPAAM/VP/PEG-A co-polymeric nanoparticles**. Please refer to text for additional details. NIPAAM = N-isopropylacrylamide; VP = N-vinyl-2-pyrrolidone (VP); PEG-A = poly(ethyleneglycol)monoacrylate; MBA = N,N'-Methylene bis acrylamide (MBA), APS = ammonium persulphate (APS); FAS = Ferrous ammonium sulphate; TEMED = Tetramethylethylenediamine.

Random co-polymerization of NIPAAM with VP and PEG-A was performed by free radical polymerization process of the micellar aggregates of the amphipilic monomers (Figure 1). The polymeric nanoparticles formed in this way also have an amphiphilic character with a hydrophobic core inside the micelles, and a hydrophilic outer shell composed of hydrated amides, pyrrolidone and PEG moieties that project from the monomeric units [24,25].

**Figure 2:**
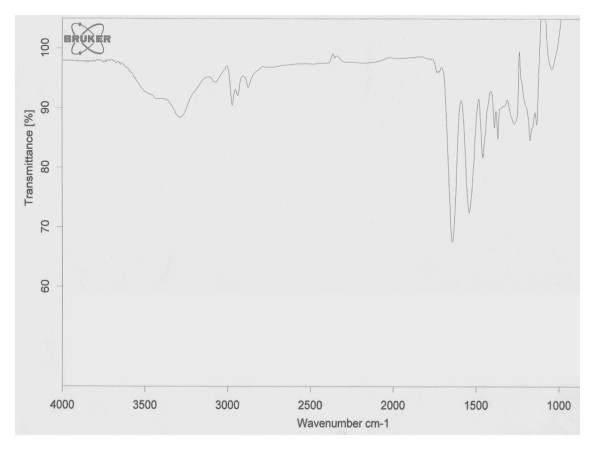
**Fourier transform infra-red (FTIR) spectrum of copolymeric nanoparticles**. The FTIR spectrum of (NIPAAM-VP-PA) copolymer demonstrates complete polymerization and absence of monomers in the physical mixture. The spectra of the three commercially available monomers are not shown.

Mid infra-red (IR) spectra of NIPAAM, VP, PEG-A, and "void" (empty) polymeric nanoparticles were obtained to determine whether appropriate polymerization has occurred or whether monomers were present in the physical mixture. As seen in Figure 2, strong peaks in the range of 800–1000 cm^-1 ^corresponding to the stretching mode of vinyl double bonds disappeared in the spectrum of polymer indicating that polymerization has taken place. The water attached in the process of hydration of the polymer and proton exchange with the solvent gives rise to a broad and intense peak at 3300 cm^-1^. The – CH- stretching vibration of the polymer backbone is manifested through peaks at 2936–2969 cm^-1^, while peaks at 1642 and 1540 cm^-1 ^correspond to the amide carbonyl group and the bending frequency of the amide N-H group respectively. The absorptions bands in the region 1443–1457 cm^-1 ^are due to the bending vibration of CH_3 _group and the bending vibration of CH_2 _group can be identified in a slightly higher region.

**Figure 3:**
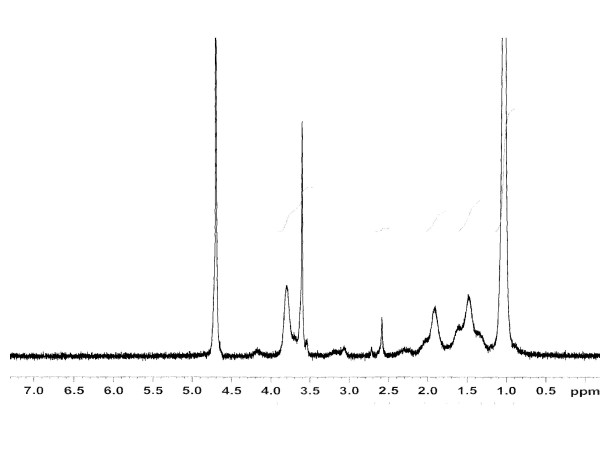
**Nuclear magnetic resonance (NMR) spectrum of copolymeric nanoparticles**. The NMR spectra further confirms the formation of the copolymer as is evident by the corresponding signal peaks of the different protons present in the polymeric backbone. The spectra of the three commercially available monomers are not shown.

In Figure 3, we illustrate the typical ^1^H-NMR spectrum and the chemical shift assignments of the monomers as well as the copolymer formed. Polymerization is indicated by the absence of the proton resonance of the vinyl end groups of the monomers in the spectrum of the formed co-polymeric micelle. Rather, resonance can be observed at the upfield region (δ = 1.4–1.9 ppm), attributable to the saturated protons of the polymeric network. The broad resonance peak at δ = 0.8–1.0 ppm are from the methyl protons of the isopropyl group. The signal peaks for the methyne proton (>CH-) of N-isopropylacrylamide group and methylene protons (-CH_2_-) of polyethylene oxide can be observed at 3.81 and 3.71 ppm respectively.

**Figure 4:**
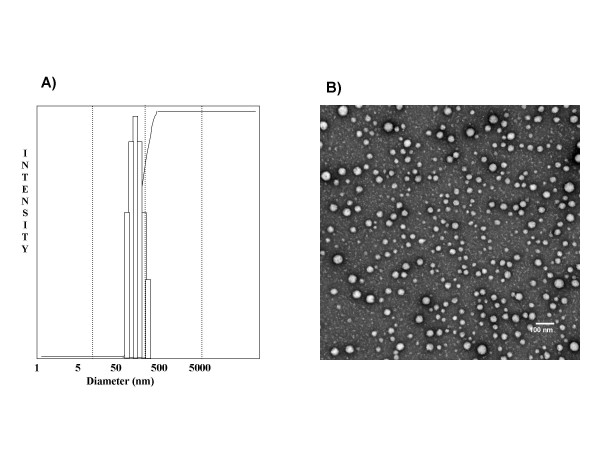
**Size characterization of the polymeric nanoparticles using dynamic laser light scattering (DLS) and transmission electron micrograph (TEM) studies**. (A) DLS of the polymeric nanoparticles confirms a narrow size distribution in the 50 nm range. All the data analysis was performed in automatic mode. Measured size was presented as the average value of 20 runs. B) TEM picture demonstrates particles with a spherical morphology, low polydispersity, and an average size of 45 nm, comparable to what is observed in the DLS studies.

The size and size distribution of the polymeric nanoparticles were measured by means of dynamic light scattering (DLS). In Figure 4A, the typical size distribution of the nanoparticles is illustrated, and the average size corresponds to less than 50 nm diameter at 25°C with a narrow size distribution. Transmission electron microscopy (TEM) of the polymeric nanoparticles is illustrated in Figure 4B, and demonstrates that the particles have spherical morphology and low polydispersity with an approximate size of around 45 nm diameter, which is comparable to the size obtained from DLS measurements.

**Figure 5:**
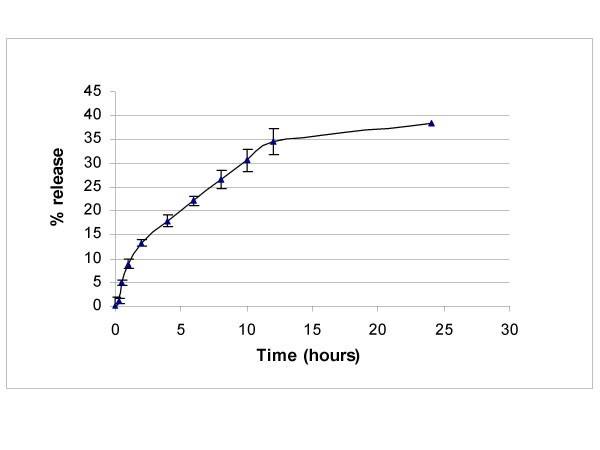
***In vitro *release kinetics of nanocurcumin**. The release kinetics of nanocurcumin demonstrates ~40% release of curcumin from the co-polymer at 24 hours, when dispersed in phosphate buffer at physiological pH. The error bars represent mean and standard deviations of experiments performed in triplicate.

The entrapment efficiency of curcumin within the nanoparticles was found to be >90%, based on calculations described in the Methods. The *in vitro *release profile of the loaded curcumin from the nanoparticles at physiological pH is illustrated in Figure 5. Curcumin release occurs in a sustained manner, such that only 40% of the total drug is releaed from the nanoparticles at 24 hours.

### In vitro and in vivo toxicity studies of void polymeric nanoparticles

**Figure 6:**
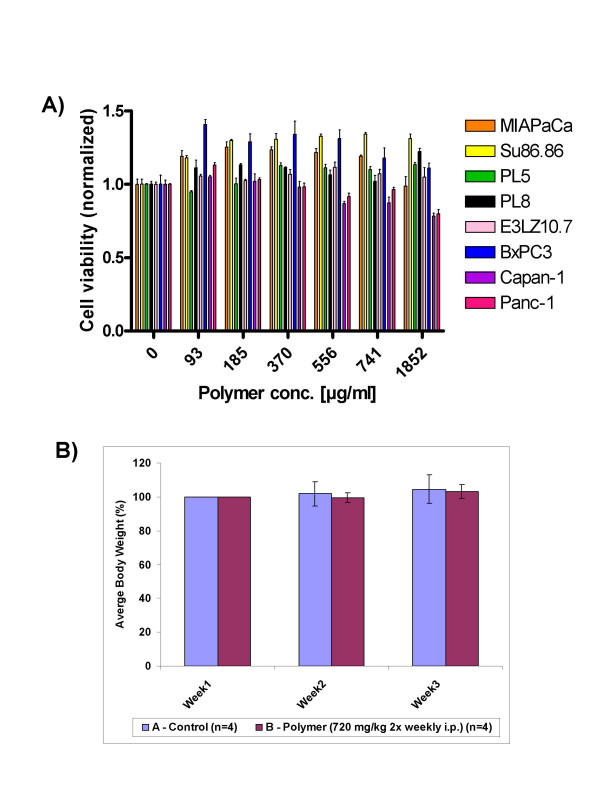
**Toxicity profile of void polymeric nanoparticles**. A) A series of eight pancreatic cancer cell lines were exposed to a 20-fold range of void polymeric nanoparticles (93 – 1852 μg/mL), and viability assays (MTT) were performed at 72 hours. Compared to vehicle treated cells, no cytotoxicity is observed in the cells exposed to polymeric nanoparticles. The error bars represent mean and standard deviations of experiments performed in triplicate. B) *In vivo *toxicity studies were performed by administration of polymeric nanoparticles (720 mg/kg intra-peritoneal twice weekly, three weeks) to a group of 4 athymic mice, which were weighed at weekly intervals in comparison to control mice (N = 4). No significant differences in body weight were seen; at necropsy, no gross toxicity was evident. The error bars represent mean and standard deviations of experiments performed in triplicate.

An ideal drug delivery platform must be biodegradable, biocompatible and not be associated with incidental adverse effects. The toxicity profile of the void polymeric nanoparticles was studied *in vitro *and *in vivo*. In a panel of eight human pancreatic cancer cell lines (Figure 6A), we found no evidence of toxicity in cell viability assays, across a 20-fold dose range of the void nanoparticles. We then studied the effects of these particles in athymic ("nude") mice, a commonly used vehicle for preclinical tumor studies. The mice were randomized to two arms of 4 mice each – control and void nanoparticles (720 mg/kg i.p. twice weekly for three weeks). As seen in Figure 6B, despite the relatively large dosage, the mice receiving void nanoparticles demonstrated no evidence of weight loss, and no gross organ changes were seen at necropsy. No behavioral changes were observed in the mice during the course of administration, or in the ensuing follow up period.

### Nanocurcumin inhibits the growth of pancreatic cancer cell lines and abrogates colony formation

**Figure 7:**
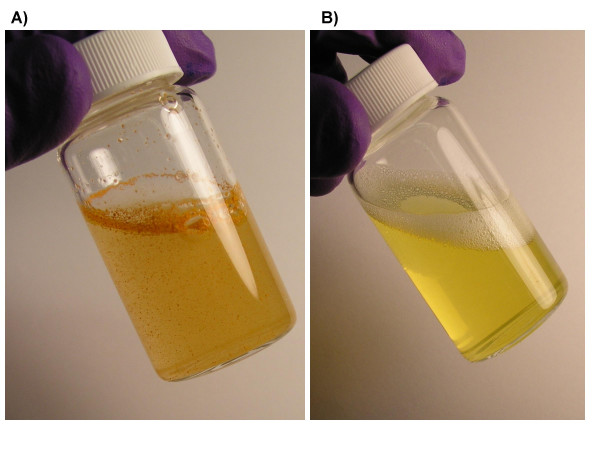
**Nano-encapsulation renders curcumin completely dispersible in aqueous media**. (a) Free curcumin is poorly soluble in aqueous media, and macroscopic flakes can be seen floating in the bottle. In contrast, the equivalent quantity of curcumin encapsulated in polymeric nanoparticles is fully dispersible in aqueous media (b).

**Figure 8:**
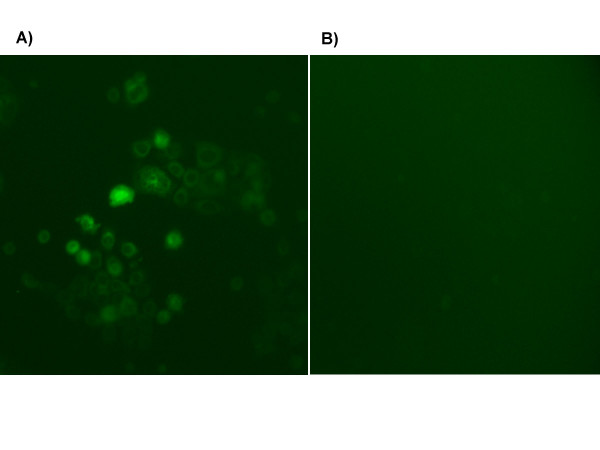
**Intracellular uptake of nanocurcumin by pancreatic cancer cell lines**. Marked increase in fluorescence was observed by fluorescent microscopy in BxPC3 cells incubated with nanocurcumin (a) as compared to untreated control cells (b), in line with cellular uptake of curcumin in (a).

**Figure 9:**
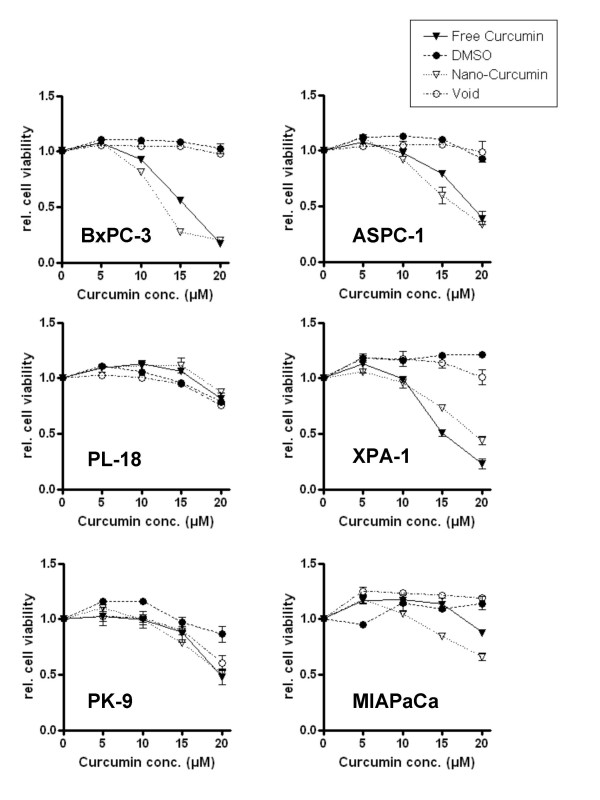
**Nanocurcumin inhibits the growth of pancreatic cancer cell lines**. Cell viability (MTT) assays were performed using equivalent dosages of free curcumin and nanocurcumin in a panel of human pancreatic cancer cell lines. The assay was terminated at 72 hours, and colorimetric determination of cell viability performed. Four of six cell lines demonstrate response to nanocurcumin (defined as an IC_50 _in the 10–15 μM range) – BxPC3, ASPC-1, PL-11 and XPA-1, while two lines are curcumin-resistant – PL-18 and PK-9. All assays were performed in triplicate, and the mean ± standard deviations are presented.

**Figure 10:**
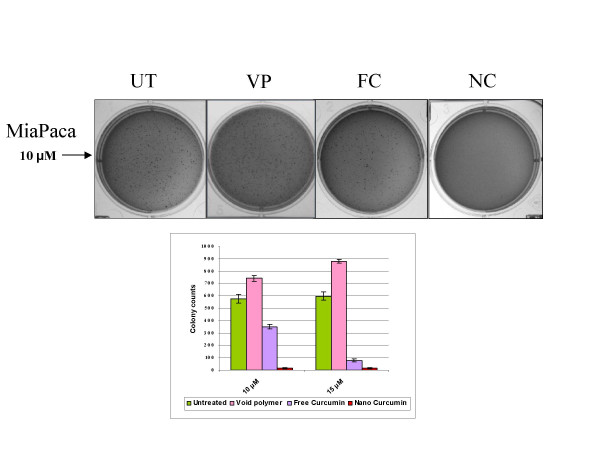
**Nanocurcumin inhibits the clonogenic potential of pancreatic cancer cell lines**. Colony assays in soft agar were performed comparing the effects of free and nanocurcumin in inhibiting the clonogenicity of the pancreatic cancer cell line, MiaPaca. Representative plates are illustrated for untreated cells (UT), void polymeric nanoparticle-treated cells (VP), free curcumin-treated cells (FC) and nanocurcumin-treated cells (NC), the last two at the equivalent of 10 μM curcumin dosage. All assays were performed in triplicates, and the mean ± standard deviations are presented.

Free curcumin is poorly soluble in aqueous media, with macroscopic undissolved flakes of the compound visible in the solution (Figure 7A); in contrast, nanocurcumin is a clear, dispersed formulation, with its hue derived from the natural color of curcumin (Figure 7B). We performed a series of *in vitro *functional assays to better characterize the anti-cancer properties of nanocurcumin, using human pancreatic cancer cells as a model system, and directly comparing its efficacy to free curcumin. The choice of the cancer type was based on multiple previous reports confirming the activity of free curcumin against pancreatic cancer cell lines [7,8,26]. As seen in Figures 8A and 8B, the polymeric nanoparticles encapsulating curcumin are robustly taken up by pancreatic cancer cells, indicated by the fluorescence emitted from the accumulated intra-cytoplasmic drug. In cell viability (MTT) assays performed against a series of pancreatic cancer lines, nanocurcumin consistently demonstrated comparable efficacy to free curcumin (Figure 9), although some cell lines were resistant to the agent *per se*. Nanocurcumin was effective in its ability to block clonogenicity of the MiaPaca pancreatic cancer cell line in soft agar assays (Figure 10). In comparison to untreated cells, or cells exposed to void polymeric nanoparticles, both free curcumin and nanocurcumin caused inhibition of clonogenicity at 10 and 15 μM dosages; the effect with nanocurcumin was somewhat more pronounced at the lower dose.

### Nanocurcumin inhibits NFκB function in pancreatic cancer cell lines and downregulates multiple pro-inflammatory cytokines

**Figure 11:**
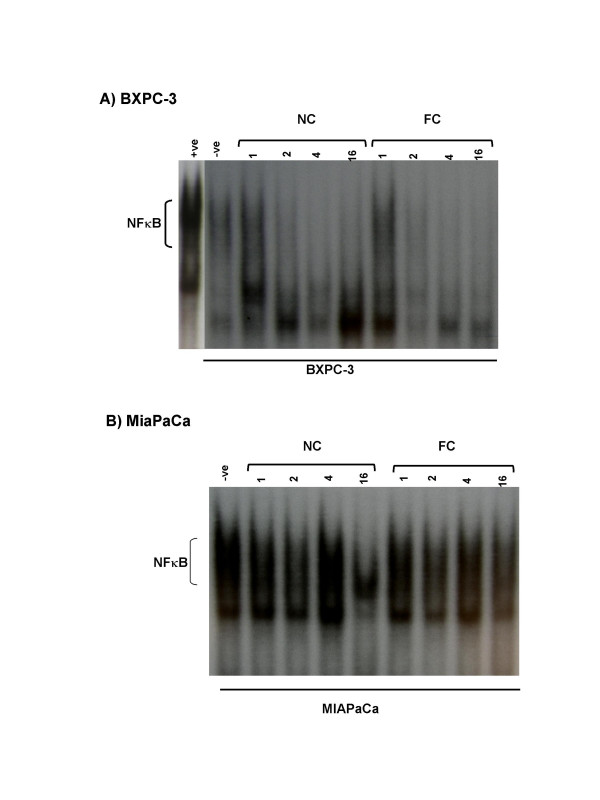
**Nanocurcumin blocks activation of nuclear factor kappa B in pancreatic cancer cell lines**. Electrophoretic mobility shift assay (EMSA) or "gel shift" assay for assessment of NFκB inhibition in pancreatic cancer cell lines. Nuclear extracts were prepared from free curcumin (FC) and nanocurcumin (NC)-treated BxPC3 and MiaPaca cells, after 1 hour, 2 hours, 4 hours and 16 hours (overnight) exposure to the respective formulation. Inhibition of NFκB function is gauged by faster migration (i.e., absence of NFκB binding) of the radio-labeled kappa-binding oligonucleotide. In BxPC3 cells, inhibition of NFκB is seen as early as 1–2 hours following curcumin exposure in both free and nanoparticulate formulations. In contrast, in MiaPaca cells, inhibition of binding and consequent gel shift is seen only after overnight incubation in the nanocurcumin-treated cells, while no discernible gel shift is apparent in the free curcumin treated cells. TPA-activated Jurkat cells were used as positive control and untreated cells as negative control.

**Figure 12:**
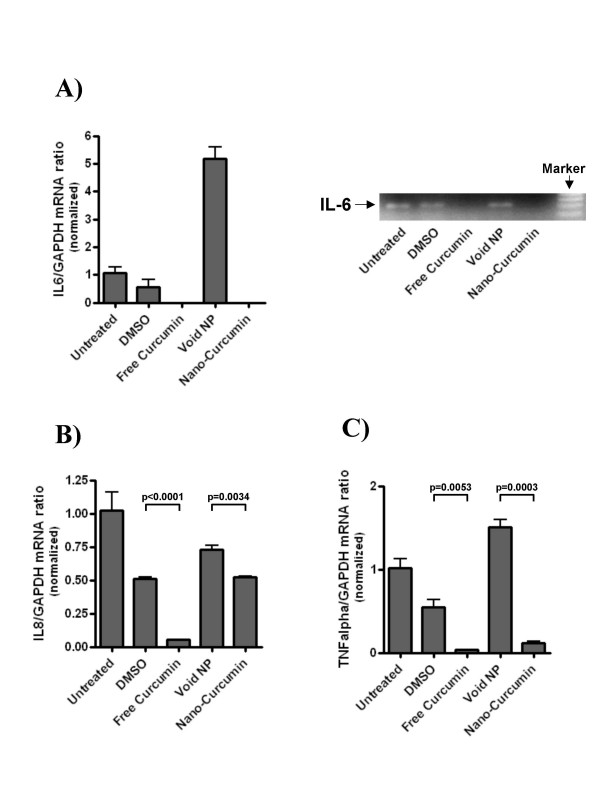
**Nanocurcumin downregulates steady state transcripts of multiple pro-inflammatory cytokines**. Peripheral blood mononuclear cells (PBMCs) were activated with phytohaemagglutinin (PHA) and 1 μg/ml lipopolysaccharide (LPS), and exposed to either free curcumin or nanocurcumin at 20 μM. Thereafter, transcript levels of three pro-inflammatory cytokines IL-6, IL-8 and TNFα were measured by real time quantitative RT-PCR analysis. For IL-6, semi-quantitative RT-PCR was also performed to confirm the real time data. IL-6 expression is completely abrogated by exposure to free curcuminand nanocurcumin (A). IL-8 levels are also reduced with nanocurcumin therapy, albeit less than in free curcumin-treated PBMCs (B), while comparable and significant knockdown of TNFα transcripts is seen with both formulations(C). The error bars represent mean and standard deviations of experiments performed in triplicate.

**Figure 13:**
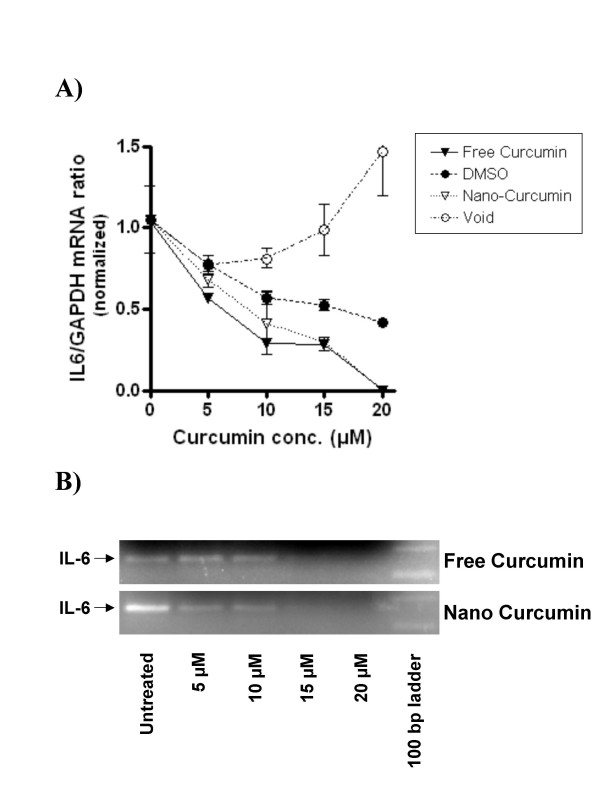
**Nanocurcumin inhibits IL-6 synthesis in a dose-dependent manner**. PBMCs of a healthy donor were incubated with PHA to stimulate T-cells, and LPS to trigger monocyte-derived cytokine production. Cells were exposed to increasing concentrations (0, 5, 10, 15 or 20 μM) of free or nanocurcumin, or equivalents amounts of DMSO or void nanoparticles, respectively for 24 h. Quantitative RT-PCR revealed dose-dependent inhibition of IL-6 mRNA synthesis by both curcumin formulations (A). Complete blockade of IL-6 transcripts was achieved by adding free or nanocurcumin at 20 μM, even in PBMC co-stimulated with PHA and LPS (B). The error bars represent mean and standard deviations of experiments performed in triplicate.

We then analyzed the mechanisms of action of nanocurcumin on pancreatic cancer cell lines, and compared the functional pathways impacted by nanocurcumin to what has been previously reported for free curcumin [26-31]. A principal cellular target of curcumin in cancer cells is activated nuclear factor kappa B (NFκB), with many of the pleiotropic effects of curcumin being ascribed to inhibition of this seminal transcription factor. In electrophoretic mobility shift ("gel shift") assays to assess for the DNA binding ability of NFκB, we demonstrate that nanocurcumin robustly inhibits NFκB function in pancreatic cancer cell lines BxPC3 and MiaPaca (Figures 11A and 11B). In BxPC3 cells, inhibition of NFκB (assessed by a shift in migration of radio-labeled p65-binding oligonucleotide) can be seen as early as 1–2 hours post exposure to both free and nanocurcumin. In MiaPaca cells, we see persistent activation of NFκB in cells exposed to free curcumin after overnight incubation, while a perceptible gel shift is observed in the nanocurcumin treated cells. Lastly, we examined whether nanocurcumin can inhibit pro-inflammatory cytokines in peripheral blood mononuclear cells (PBMCs); many of these cytokines (IL-6, IL-8, and TNFα) have also been implicated in the carcinogenesis process, including the induction of angiogenesis [32]. Incubation of stimulated PBMCs with both free and nanocurcumin decreased steady-state mRNA levels of IL-6, IL-8 and TNFα, compared to DMSO and void nanoparticle-treated cells (Figure 12A–C), with evidence of dose dependent reduction of IL-6 by both agents (Figures 13A–B).

## Conclusion

In the course of the past decade, the field of drug delivery has been revolutionized with the advent of nanotechnology, wherein biocompatible nanoparticles have been developed as inert systemic carriers for therapeutic compounds to target cells and tissues [33-38]. A recent example of the impact of nanomedicine in drug delivery is underscored by the success of Abraxane™, an albumin nanoparticle conjugate of paclitaxel, and the first FDA-approved anti-cancer agent in this emerging class of drug formulations [39]. In a quest for developing stable and efficient systemic carriers for hydrophobic anti-cancer compounds, our laboratory has developed cross-linked polymeric nanoparticles comprised of N-isopropylacrylamide (NIPAAM), N-vinyl-2-pyrrolidinone (VP) and poly(ethyleneglycol) acrylate (PEG-A). We demonstrate the essential non-toxicity of the void polymeric formulation *in vitro *and *in vivo*, underscoring the potential of these nanoparticles as a carrier for hydrophobic drugs.

Peer reviewed publications numbering in the 100 s have reiterated the potency of curcumin against a plethora of human cancer lines in the laboratory (selected reviews include [1,4-6,40,41]). Equally important, free curcumin was shown *not *to be cytotoxic to normal cells, including hepatocytes, mammary epithelial cells, kidney epithelial cells, lymphocytes, and fibroblasts at the dosages required for therapeutic efficacy against cancer cell lines [42-46]; these *in vitro *findings are underscored by the limited human clinical trials performed with oral curcumin, wherein doses up to 10 grams per day have had minimal adverse effects, even to the highly exposed gastrointestinal mucosa [18-22]. Nevertheless, few clinical trials have been performed with this agent.

A liposomal curcumin formulation was recently described that demonstrates comparable potency to free curcumin, and which can be administered via the parenteral route [47]. Even as further studies with this liposomal formulation are awaited, it is emphasized that liposomes, which are metastable aggregates of lipids, tend to be more heterogeneous, and larger in size (typically 100–200 nm) than most nanoparticles. We have synthesized a nanoparticulate formulation of curcumin – nanocurcumin – wherein the polymeric nanoparticles formed are consistently less than 100 nm in size (mostly in the 50 nm size range), as stated in the National Nanotechnology Initiative's (NNI's) definition of "nanomaterials". We have demonstrated that our nanocurcumin formulation has comparable efficacy to free curcumin against pancreatic cancer cell lines *in vitro*, by inhibiting cell viability and colony formation in soft agar. Further, our studies confirm that nanocurcumin retains the mechanistic specificity of free curcumin, inhibiting the activation of the seminal transcription factor NFκB, and reducing steady state levels of pro-inflammatory cytokines like interleukins and TNFα.

Nanocurcumin opens up avenues for systemic therapy of human cancers, as well as other human maladies like Alzheimer disease [48-51] and cystic fibrosis [52-54], wherein the beneficial effects of curcumin have been propounded. Future studies using relevant experimental models will enable addressing these scenarios in an *in vivo *setting, and should facilitate the eventual clinical translation of this well known but under-utilized therapeutic agent.

## Methods

### Preparation of polymeric nanoparticles

A co-polymer of N-isopropylacrylamide (NIPAAM) with N-vinyl-2-pyrrolidone (VP) poly(ethyleneglycol) monoacrylate (PEG-A) was synthesized through free radical polymerization as shown in the accompanying flowchart (Figure 1). NIPAAM, VP and PEG-A were obtained from Sigma chemicals (St. Louis, MO). NIPAAM was recrystallized using hexane, VP was freshly distilled before use, and PEG-A was washed with n-hexane three times to remove any inhibitors; Millipore water and other chemicals were used as-is. Thereafter, the water-soluble monomers – NIPAAM, VP and PEG-A were dissolved in water in 90: 5: 5 molar ratios. The polymerization was initiated using ammonium persulphate (APS, Sigma) as an initiator in a nitrogen (N_2_) atmosphere. Ferrous ammonium sulphate (FAS, Sigma) was added to activate the polymerization reaction, and also to ensure complete polymerization of the monomers. In a typical experimental protocol, 90 mg NIPAAM, 5 μl freshly distilled VP, and 500 μl PEG-A (1% w/v) were added in 10 ml of water. To cross-link the polymer chains, 30 μl of N,N'-Methylene bis acrylamide (MBA, Sigma, 0.049 g/ml) was added to the aqueous solution of monomers. The dissolved oxygen was removed by passing nitrogen gas for 30 minutes. Thereafter, 20 μl of FAS (0.5% w/v), 30 μl of APS and 20 μl of TEMED (Invitrogen, Carlsbad CA, USA) were added to initiate the polymerization reaction. The polymerization was performed at 30°C for 24 hours in a N_2 _atmosphere. After the polymerization was complete, the total aqueous solution of polymer was dialyzed overnight using a Spectrapore^® ^membrane dialysis bag (12 kD cut off) to remove any residual monomers. The dialyzed solution was then lyophilized immediately to obtain a dry powder for subsequent use, which was easily re-dispersible in aqueous media. The yield of the polymeric nanoparticles was typically more than 90% with this protocol.

### Loading of curcumin

Curcumin was a kind gift of Indsaff, Inc. (Batala, Punjab, India). Curcumin loading in the polymeric nanoparticles was done by using a post-polymerization method. In this process of loading, the drug is dissolved after the co-polymer formation has taken place. The physical entrapment of curcumin in NIPAAM/VP/PEG-A polymeric nanoparticles was carried out as follows: 100 mg of the lyophilized powder was dispersed in 10 ml distilled water and was stirred to re-constitute the micelles. Free curcumin was dissolved in chloroform (CHCl_3_; 10 mg/ml) and the drug solution in CHCl_3 _was added to the polymeric solution slowly with constant vortexing and mild sonication. Curcumin is directly loaded into the hydrophobic core of nanoparticles by physical entrapment. The drug-loaded nanoparticles are then lyophilized to dry powder for subsequent use.

### Entrapment efficiency (E %)

The entrapment efficiency (*E *%) of curcumin loaded in NIPAAM-VP-PEG-A nanoparticles was determined as follows: the nanoparticles were separated from the un-entrapped free drug using NANOSEP (100 kD cut off) membrane filter and the amount of free drug in the filtrate was measured spectrophotometrically using a WALLAC plate reader at 450 nm. The E% was calculated by

E% = ([Drug]_tot _- [Drug]_free_)/[Drug]_tot _× 100

### Fourier Transform Infrared (FT-IR) studies of polymeric nanoparticles

Mid infra red (IR) spectrum of NIPAAM, VP and PEG-A monomers, as well as the void polymeric nanoparticles were taken using Bruker Tensor 27 (FT-IR) spectrophotometer (Bruker Optics Inc., Billerica, MA, USA).

### ^1-^H Nuclear Magnetic Resonance (NMR) studies

The NMR spectrum of monomers NIPAAM, VP and PA, as well as void polymeric nanoparticles were taken by dissolving the samples in D_2_O as solvent using Bruker Avance 400 MHz spectrometer (Bruker BioSpin Corporation, Billerica, MA, USA).

### Dynamic light scattering (DLS) measurements

DLS measurements for determining the average size and size distribution of the polymeric micelles were performed using a Nanosizer 90 ZS (Malvern Instruments, Southborough, MA). The intensity of scattered light was detected at 90° to an incident beam. The freeze-dried powder was dispersed in aqueous buffer and measurements were done, after the aqueous micellar solution was filtered with a microfilter having an average pore size of 0.2 mm (Millipore). All the data analysis was performed in automatic mode. Measured size was presented as the average value of 20 runs, with triplicate measurements within each run.

### Transmission electron microscopy (TEM)

TEM pictures of polymeric nanoparticles were taken in a Hitachi H7600 TEM instrument operating at magnification of 80 kV with 1 K × 1 K digital images captured using an AMT CCD camera. Briefly, a drop of aqueous solution of lyophilized powder (5 mg/ml) was placed on a membrane coated grid surface with a filter paper (Whatman No. 1). A drop of 1% uranyl acetate as immediately added to the surface of the carbon coated grid. After 1 min excess fluid was removed and the grid surface was air dried at room temperature before loaded in the microscope.

### In vitro release kinetics of nanocurcumin

A known amount of lyophilized polymeric nanoparticles (100 mg) encapsulating curcumin was dispersed in 10 ml phosphate buffer, pH 7.4, and the solution was divided in 20 microfuge tubes (500 μl each). The tubes were kept in a thermostable water bath set at room temperature. Free curcumin is completely insoluble in water; therefore, at predetermined intervals of time, the solution was centrifuged at 3000 rpm for 10 minutes to separate the released (pelleted) curcumin from the loaded nanoparticles. The released curcumin was redissolved in 1 ml of ethanol and the absorbance was measured spectrophotometrically at 450 nm. The concentration of the released curcumin was then calculated using standard curve of curcumin in ethanol. The percentage of curcumin released was determined from the equation

Release (%)=[Curcumin]rel[Curcumin]tot×100
 MathType@MTEF@5@5@+=feaafiart1ev1aaatCvAUfKttLearuWrP9MDH5MBPbIqV92AaeXatLxBI9gBaebbnrfifHhDYfgasaacH8akY=wiFfYdH8Gipec8Eeeu0xXdbba9frFj0=OqFfea0dXdd9vqai=hGuQ8kuc9pgc9s8qqaq=dirpe0xb9q8qiLsFr0=vr0=vr0dc8meaabaqaciaacaGaaeqabaqabeGadaaakeaacqqGsbGucqqGLbqzcqqGSbaBcqqGLbqzcqqGHbqycqqGZbWCcqqGLbqzcqqGGaaicqGGOaakcqGGLaqjcqGGPaqkcqGH9aqpdaWcaaqaaiabcUfaBjabboeadjabbwha1jabbkhaYjabbogaJjabbwha1jabb2gaTjabbMgaPjabb6gaUjabc2faDnaaBaaaleaacqqGYbGCcqqGLbqzcqqGSbaBaeqaaaGcbaGaei4waSLaee4qamKaeeyDauNaeeOCaiNaee4yamMaeeyDauNaeeyBa0MaeeyAaKMaeeOBa4Maeiyxa01aaSbaaSqaaiabbsha0jabb+gaVjabbsha0bqabaaaaOGaey41aqRaeGymaeJaeGimaaJaeGimaadaaa@6275@

where, [Curcumin]_rel _is the concentration of released curcumin collected at time *t *and [Curcumin]_tot _is the total amount of curcumin entrapped in the nanoparticles.

### In vitro and vivo toxicity studies with void polymeric nanoparticles

In order to exclude the possibility of *de novo *toxicity from the polymeric constituents, we utilized void nanoparticles against a panel of eight human pancreatic cancer cell lines (MiaPaca2, Su86.86, BxPC3, Capan1, Panc1, E3LZ10.7, PL5 and PL8). These cells were exposed to void nanoparticles for 96 hours across a 20-fold concentration range (93 – 1852 μg/mL) and cell viability measured by MTS assay, as described below. Further, limited *in vivo *toxicity studies were performed in athymic (nude) mice by intraperitoneal injection of void polymeric nanoparticles at a considerably high dosage of 720 mg/kg twice weekly, for a period of three weeks. Mice receiving intra-peritoneal nanocurcumin (N = 4) were weighed weekly during the course of therapy and average weight compared to that of control littermate nude mice (N = 4). At the culmination of the three week course, mice were euthanized and necropsy performed to exclude any intraperitoneal deposition of polymers, or gross organ toxicities.

### Fluorescence microscopy for nanocurcumin uptake by pancreatic cancer cells

Curcumin is naturally fluorescent in the visible green spectrum. In order to study uptake of curcumin encapsulated in nanoparticles, BxPC3 cells were plated in 100 mm dishes, and allowed to grow to sub-confluent levels. Thereafter, the cells were incubated with nanocurcumin for 2–4 hours, and visualized in the FITC channel.

### Cell viability (MTS) assays in pancreatic cancer cell lines exposed to nanocurcumin

Growth inhibition was measured using the CellTiter 96^® ^A_queous _Cell Proliferation Assay (Promega), which relies on the conversion of a tetrazolium compound (MTS) to a colored formazan product by the activity of living cells. Briefly, 2000 cells/well were plated in 96 well plates, and were treated with 0, 5, 10, 15 and 20 μM concentrations of free curcumin and equivalent nanocurcumin, for 72 hours, at which point the assay was terminated, and relative growth inhibition compared to vehicle-treated cells measured using the CellTiter 96^® ^reagent, as described in the manufacturer's protocol. A panel of ten human pancreatic cancer cell lines were examined (BxPC3, AsPC1, MiaPaca, XPA-1, XPA-2, PL-11, PL-12, PL-18, PK-9 and Panc 2.03) in the MTT assays; the sources and culture conditions of these ten lines have been previously described [58]. All experiments were set up in triplicates to determine means and standard deviations.

### Colony assays in soft agar

Colony formation in soft agar was assessed for therapy with free curcumin and equivalent dosage of nanocurcumin. Briefly, 2 ml of mixture of serum supplemented media and 1 % agar containing 5, 10 or 15 μM of free curcumin and equivalent nanocurcumin was added in a 35 mm culture dish and allowed to solidify (base agar) respectively. Next, on top of the base layer was added a mixture of serum supplemented media and 0.7 % agar (total 2 mL) containing 10,000 MiaPaca2 cells in the presence of void polymer, free or nano-curcumin, and was allowed to solidify (top agar); a fourth set of plates contained MiaPac2 cells without any additives. Subsequently, the dishes were kept in tissue culture incubator maintained at 37°C and 5 % CO_2 _for 14 days to allow for colony growth. All assays were performed in triplicates. The colony assay was terminated at day 14, when plates were stained and colonies counted on ChemiDoc XRS instrument (Bio-Rad, Hercules, CA).

### Electrophoretic mobility shift assay (EMSA)

Nuclear extracts were prepared as described [59]. Briefly, double-stranded oligonucleotides containing a consensus binding site for c-Rel (5'-GGG GAC TTT CCC-3') (Santa Cruz Biotechnology) were 5' end-labeled using polynucleotide kinase and [^32^P]dATP. Nuclear extracts (2.5–5 μg) were incubated with ≈1 μl of labeled oligonucleotide (20,000 c.p.m.) in 20 μl of incubation buffer (10 mM Tris-HCl, 40 mM NaCl, 1 mM EDTA, 1 mM β-mercaptoethanol, 2% glycerol, 1–2 μg of poly dI-dC) for 20 min at 25°C. DNA-protein complexes were resolved by electrophoresis in 5% non-denaturing polyacrylamide gels and analyzed by autoradiography.

### Determination of IL-6, IL-8 and TNF-alpha synthesis

IL-6, IL-8 and TNF-alpha mRNA levels were assessed as described previously [55]. Briefly, peripheral blood mononuclear cells (PBMC) from a healthy donor were isolated by centrifugation on a Ficoll Hypaque density gradient (GE Healthcare Biosciences) and washed twice with phosphate buffered saline (PBS; Invitrogen, Carlsbad, CA). Next, 500,000 cells per well of a 24 well plate in 1 ml of RPMI (Invitrogen) supplemented with 10% FBS (Invitrogen) and 1× Pen/Strep (Biofluids, Camarillo, CA) were co-stimulated with 2% phytohaemagglutinin (PHA M-Form, liquid; Invitrogen) and 1 μg/ml lipopolysaccharide (LPS; Sigma-Aldrich, St. Louis, MO) in the presence of free or nanocurcumin, using solvent and void nanoparticles as controls, respectively. Cells were lysed after 24 hours incubation at 37°C and 5% CO_2 _and RNA extracted using the RNeasy Mini Kit (Qiagen, Valencia, CA). Relative fold steady-state mRNA levels were determined on a 7300 Real time PCR System (Applied Biosystems, Foster City, CA,) by RT-PCR as described [56].

## Abbreviations

N-isopropylacrylamide = NIPAAM; N-vinyl-2-pyrrolidone = VP; poly (ethyleneglycol) monoacrylate = PEG-A; nuclear factor kappa B = NFκB

## Competing interests

The author(s) declare that they have no competing interests.

## Authors' contributions

SB and SS synthesized nanocurcumin, SB, GF and CK performed the in vitro functional assays, SB and RR performed the EMSA studies, ANM and AM conceived the idea of nanocurcumin, guided the conduct of studies, supervised data analysis, and authored the manuscript.

## References

[B1] Shishodia S, Sethi G, Aggarwal BB (2005). Curcumin: getting back to the roots. Ann N Y Acad Sci.

[B2] Mohandas KM, Desai DC (1999). Epidemiology of digestive tract cancers in India. V. Large and small bowel. Indian J Gastroenterol.

[B3] Sinha R, Anderson DE, McDonald SS, Greenwald P (2003). Cancer risk and diet in India. J Postgrad Med.

[B4] Maheshwari RK, Singh AK, Gaddipati J, Srimal RC (2006). Multiple biological activities of curcumin: a short review. Life Sci.

[B5] Duvoix A, Blasius R, Delhalle S, Schnekenburger M, Morceau F, Henry E, Dicato M, Diederich M (2005). Chemopreventive and therapeutic effects of curcumin. Cancer Lett.

[B6] Aggarwal BB, Kumar A, Bharti AC (2003). Anticancer potential of curcumin: preclinical and clinical studies. Anticancer Res.

[B7] Wang Z, Zhang Y, Banerjee S, Li Y, Sarkar FH (2006). Notch-1 down-regulation by curcumin is associated with the inhibition of cell growth and the induction of apoptosis in pancreatic cancer cells. Cancer.

[B8] Lev-Ari S, Zinger H, Kazanov D, Yona D, Ben-Yosef R, Starr A, Figer A, Arber N (2005). Curcumin synergistically potentiates the growth inhibitory and pro-apoptotic effects of celecoxib in pancreatic adenocarcinoma cells. Biomed Pharmacother.

[B9] Aggarwal BB, Shishodia S, Takada Y, Banerjee S, Newman RA, Bueso-Ramos CE, Price JE (2005). Curcumin suppresses the paclitaxel-induced nuclear factor-kappaB pathway in breast cancer cells and inhibits lung metastasis of human breast cancer in nude mice. Clin Cancer Res.

[B10] Khor TO, Keum YS, Lin W, Kim JH, Hu R, Shen G, Xu C, Gopalakrishnan A, Reddy B, Zheng X (2006). Combined inhibitory effects of curcumin and phenethyl isothiocyanate on the growth of human PC-3 prostate xenografts in immunodeficient mice. Cancer Res.

[B11] Deeb D, Jiang H, Gao X, Hafner MS, Wong H, Divine G, Chapman RA, Dulchavsky SA, Gautam SC (2004). Curcumin sensitizes prostate cancer cells to tumor necrosis factor-related apoptosis-inducing ligand/Apo2L by inhibiting nuclear factor-kappaB through suppression of IkappaBalpha phosphorylation. Mol Cancer Ther.

[B12] Lev-Ari S, Strier L, Kazanov D, Madar-Shapiro L, Dvory-Sobol H, Pinchuk I, Marian B, Lichtenberg D, Arber N (2005). Celecoxib and curcumin synergistically inhibit the growth of colorectal cancer cells. Clin Cancer Res.

[B13] Inano H, Onoda M, Inafuku N, Kubota M, Kamada Y, Osawa T, Kobayashi H, Wakabayashi K (1999). Chemoprevention by curcumin during the promotion stage of tumorigenesis of mammary gland in rats irradiated with gamma-rays. Carcinogenesis.

[B14] Chuang SE, Kuo ML, Hsu CH, Chen CR, Lin JK, Lai GM, Hsieh CY, Cheng AL (2000). Curcumin-containing diet inhibits diethylnitrosamine-induced murine hepatocarcinogenesis. Carcinogenesis.

[B15] Singh SV, Hu X, Srivastava SK, Singh M, Xia H, Orchard JL, Zaren HA (1998). Mechanism of inhibition of benzo[a]pyrene-induced forestomach cancer in mice by dietary curcumin. Carcinogenesis.

[B16] Li N, Chen X, Liao J, Yang G, Wang S, Josephson Y, Han C, Chen J, Huang MT, Yang CS (2002). Inhibition of 7,12-dimethylbenz[a]anthracene (DMBA)-induced oral carcinogenesis in hamsters by tea and curcumin. Carcinogenesis.

[B17] Kawamori T, Lubet R, Steele VE, Kelloff GJ, Kaskey RB, Rao CV, Reddy BS (1999). Chemopreventive effect of curcumin, a naturally occurring anti-inflammatory agent, during the promotion/progression stages of colon cancer. Cancer Res.

[B18] Cruz-Correa M, Shoskes DA, Sanchez P, Zhao R, Hylind LM, Wexner SD, Giardiello FM (2006). Combination treatment with curcumin and quercetin of adenomas in familial adenomatous polyposis. Clin Gastroenterol Hepatol.

[B19] Sharma RA, McLelland HR, Hill KA, Ireson CR, Euden SA, Manson MM, Pirmohamed M, Marnett LJ, Gescher AJ, Steward WP (2001). Pharmacodynamic and pharmacokinetic study of oral Curcuma extract in patients with colorectal cancer. Clin Cancer Res.

[B20] Sharma RA, Euden SA, Platton SL, Cooke DN, Shafayat A, Hewitt HR, Marczylo TH, Morgan B, Hemingway D, Plummer SM (2004). Phase I clinical trial of oral curcumin: biomarkers of systemic activity and compliance. Clin Cancer Res.

[B21] Garcea G, Jones DJ, Singh R, Dennison AR, Farmer PB, Sharma RA, Steward WP, Gescher AJ, Berry DP (2004). Detection of curcumin and its metabolites in hepatic tissue and portal blood of patients following oral administration. Br J Cancer.

[B22] Cheng AL, Hsu CH, Lin JK, Hsu MM, Ho YF, Shen TS, Ko JY, Lin JT, Lin BR, Ming-Shiang W (2001). Phase I clinical trial of curcumin, a chemopreventive agent, in patients with high-risk or pre-malignant lesions. Anticancer Res.

[B23] Lao CD, Ruffin MTt, Normolle D, Heath DD, Murray SI, Bailey JM, Boggs ME, Crowell J, Rock CL, Brenner DE (2006). Dose escalation of a curcuminoid formulation. BMC Complement Altern Med.

[B24] Soni S, Babbar AK, Sharma RK, Maitra A (2006). Delivery of hydrophobised 5-fluorouracil derivative to brain tissue through intravenous route using surface modified nanogels. J Drug Target.

[B25] Tyagi R, Lala S, Verma AK, Nandy AK, Mahato SB, Maitra A, Basu MK (2005). Targeted delivery of arjunglucoside I using surface hydrophilic and hydrophobic nanocarriers to combat experimental leishmaniasis. J Drug Target.

[B26] Li L, Aggarwal BB, Shishodia S, Abbruzzese J, Kurzrock R (2004). Nuclear factor-kappaB and IkappaB kinase are constitutively active in human pancreatic cells, and their down-regulation by curcumin (diferuloylmethane) is associated with the suppression of proliferation and the induction of apoptosis. Cancer.

[B27] Aggarwal BB, Shishodia S (2004). Suppression of the nuclear factor-kappaB activation pathway by spice-derived phytochemicals: reasoning for seasoning. Ann N Y Acad Sci.

[B28] Aggarwal S, Ichikawa H, Takada Y, Sandur SK, Shishodia S, Aggarwal BB (2006). Curcumin (diferuloylmethane) down-regulates expression of cell proliferation and antiapoptotic and metastatic gene products through suppression of IkappaBalpha kinase and Akt activation. Mol Pharmacol.

[B29] Hidaka H, Ishiko T, Furuhashi T, Kamohara H, Suzuki S, Miyazaki M, Ikeda O, Mita S, Setoguchi T, Ogawa M (2002). Curcumin inhibits interleukin 8 production and enhances interleukin 8 receptor expression on the cell surface:impact on human pancreatic carcinoma cell growth by autocrine regulation. Cancer.

[B30] Shishodia S, Amin HM, Lai R, Aggarwal BB (2005). Curcumin (diferuloylmethane) inhibits constitutive NF-kappaB activation, induces G1/S arrest, suppresses proliferation, and induces apoptosis in mantle cell lymphoma. Biochem Pharmacol.

[B31] Bharti AC, Donato N, Aggarwal BB (2003). Curcumin (diferuloylmethane) inhibits constitutive and IL-6-inducible STAT3 phosphorylation in human multiple myeloma cells. J Immunol.

[B32] Ferrara N (2000). Vascular endothelial growth factor and the regulation of angiogenesis. Recent Prog Horm Res.

[B33] van Vlerken LE, Amiji MM (2006). Multi-functional polymeric nanoparticles for tumour-targeted drug delivery. Expert Opin Drug Deliv.

[B34] Torchilin VP (2007). Micellar nanocarriers: pharmaceutical perspectives. Pharm Res.

[B35] Gaucher G, Dufresne MH, Sant VP, Kang N, Maysinger D, Leroux JC (2005). Block copolymer micelles: preparation, characterization and application in drug delivery. J Control Release.

[B36] Torchilin VP (2004). Targeted polymeric micelles for delivery of poorly soluble drugs. Cell Mol Life Sci.

[B37] Kwon GS (2003). Polymeric micelles for delivery of poorly water-soluble compounds. Crit Rev Ther Drug Carrier Syst.

[B38] Panyam J, Labhasetwar V (2003). Biodegradable nanoparticles for drug and gene delivery to cells and tissue. Adv Drug Deliv Rev.

[B39] Gradishar WJ (2006). Albumin-bound paclitaxel: a next-generation taxane. Expert Opin Pharmacother.

[B40] Singh S, Khar A (2006). Biological effects of curcumin and its role in cancer chemoprevention and therapy. Anticancer Agents Med Chem.

[B41] Aggarwal BB, Shishodia S (2006). Molecular targets of dietary agents for prevention and therapy of cancer. Biochem Pharmacol.

[B42] Choudhuri T, Pal S, Das T, Sa G (2005). Curcumin selectively induces apoptosis in deregulated cyclin D1-expressed cells at G2 phase of cell cycle in a p53-dependent manner. J Biol Chem.

[B43] Tong QS, Zheng LD, Lu P, Jiang FC, Chen FM, Zeng FQ, Wang L, Dong JH (2006). Apoptosis-inducing effects of curcumin derivatives in human bladder cancer cells. Anticancer Drugs.

[B44] Ramachandran C, You W (1999). Differential sensitivity of human mammary epithelial and breast carcinoma cell lines to curcumin. Breast Cancer Res Treat.

[B45] Syng-Ai C, Kumari AL, Khar A (2004). Effect of curcumin on normal and tumor cells: role of glutathione and bcl-2. Mol Cancer Ther.

[B46] Jiang MC, Yang-Yen HF, Yen JJ, Lin JK (1996). Curcumin induces apoptosis in immortalized NIH 3T3 and malignant cancer cell lines. Nutr Cancer.

[B47] Li L, Braiteh FS, Kurzrock R (2005). Liposome-encapsulated curcumin: in vitro and in vivo effects on proliferation, apoptosis, signaling, and angiogenesis. Cancer.

[B48] Lim GP, Chu T, Yang F, Beech W, Frautschy SA, Cole GM (2001). The curry spice curcumin reduces oxidative damage and amyloid pathology in an Alzheimer transgenic mouse. J Neurosci.

[B49] Park SY, Kim DS (2002). Discovery of natural products from Curcuma longa that protect cells from beta-amyloid insult: a drug discovery effort against Alzheimer's disease. J Nat Prod.

[B50] Ono K, Hasegawa K, Naiki H, Yamada M (2004). Curcumin has potent anti-amyloidogenic effects for Alzheimer's beta-amyloid fibrils in vitro. J Neurosci Res.

[B51] Yang F, Lim GP, Begum AN, Ubeda OJ, Simmons MR, Ambegaokar SS, Chen PP, Kayed R, Glabe CG, Frautschy SA, Cole GM (2005). Curcumin inhibits formation of amyloid beta oligomers and fibrils, binds plaques, and reduces amyloid in vivo. J Biol Chem.

[B52] Egan ME, Pearson M, Weiner SA, Rajendran V, Rubin D, Glockner-Pagel J, Canny S, Du K, Lukacs GL, Caplan MJ (2004). Curcumin, a major constituent of turmeric, corrects cystic fibrosis defects. Science.

[B53] Lipecka J, Norez C, Bensalem N, Baudouin-Legros M, Planelles G, Becq F, Edelman A, Davezac N (2006). Rescue of DeltaF508-CFTR (cystic fibrosis transmembrane conductance regulator) by curcumin: involvement of the keratin 18 network. J Pharmacol Exp Ther.

[B54] Davis PB, Drumm ML (2004). Some like it hot: curcumin and CFTR. Trends Mol Med.

[B55] Feldmann G, Beaty RM, Hruban RH, Maitra A (2006). Molecular genetics of pancreatic intraepithelial neoplasia. J Hepatobiliary Pancreat Surg.

[B56] Karikari CA, Mullendore M, Eshleman JR, Argani P, Leoni LM, Chattopadhyay S, Hidalgo M, Maitra A (2005). Homozygous deletions of methylthioadenosine phosphorylase in human biliary tract cancers. Mol Cancer Ther.

